# Dosing Cefazolin for Surgical Site Infection Prophylaxis in Adolescent Idiopathic Scoliosis Surgery: Intermittent Bolus or Continuous Infusion?—A Pilot Study

**DOI:** 10.3390/jcm13123524

**Published:** 2024-06-16

**Authors:** Yichao Yu, F. Cole Dooley, Anna Woods, Amy Gunnett, Hardik Chandasana, Elham Amini, Cynthia Garvan, Stephanie Ihnow, Laurel C. Blakemore, Taran Sangari, Christoph N. Seubert

**Affiliations:** 1 Department of Pharmaceutics, University of Florida, Gainesville, FL 32610, USAhardik.x.chandasana@gsk.com (H.C.); elham.amoni@gilead.com (E.A.); 2 Drug Development, Clinical Pharmacology, Boehringer Ingelheim, Ridgefield, CT 06810, USA; 3 Department of Anesthesiology, University of Florida College of Medicine, Gainesville, FL 32610, USA; 4 Clinical Pharmacology, Modeling, and Simulation, Glaxo-Smith-Kline, Collegeville, PA 19426, USA; 5 Department of Clinical Pharmacology, Gilead Sciences, Foster City, CA 94404, USA; 6 Department of Orthopaedic Surgery & Sports Medicine, University of Florida College of Medicine, Gainesville 32610, FL, USA; 7 Pediatric Specialists of Virginia, Fairfax, VA 22031, USA; 8 Department of Anesthesiology, University of California at San Francisco, Oakland, CA 94609, USA

**Keywords:** scoliosis, spinal fusion, cefazolin, surgical wound infection\prevention and control, microdialysis

## Abstract

**Background:** Cefazolin may minimize the risk of surgical site infection (SSI) following posterior spinal fusion (PSF) for adolescent idiopathic scoliosis (AIS). Cefazolin dosing recommendations vary and there is limited evidence for achieved tissue concentrations. **Methods:** We performed a randomized, controlled, prospective pharmacokinetic pilot study of 12 patients given cefazolin by either intermittent bolus (30 mg/kg every 3 h) or continuous infusion (30 mg/kg bolus followed by 10/mg/kg per hour) during PSF for AIS. **Results:** Patients were well matched for demographic and perioperative variables. While total drug exposure, measured as area-under-the-curve (AUC), was similar in plasma for bolus and infusion dosing, infusion dosing achieved greater cefazolin exposure in subcutaneous and muscle tissue. Using the pharmacodynamic metric of time spent above minimal inhibitory concentration (MIC), both bolus and infusion dosing performed well. However, when targeting a bactericidal concentration of 32 µg/mL, patients in the bolus group spent a median of 1/5 and 1/3 of the typical 6 h operative time below target in subcutaneous and muscle tissue, respectively. **Conclusions:** We conclude that intraoperative determination of cefazolin tissue concentrations is feasible and both bolus and infusion dosing of cefazolin achieve concentrations in excess of typical MICs. Infusion dosing appears to more consistently achieve bactericidal concentrations in subcutaneous and muscle tissues.

## 1. Introduction

Adolescent idiopathic scoliosis (AIS) is a curvature of the spine not caused by a vertebral defect, neurologic disease, or muscular disease. Progression of the curve frequently occurs during the adolescent growth spurt, and skeletal maturity is an important factor affecting treatment approaches [1]. The severity of the curve is assessed by the Cobb angle in the frontal/coronal plane on a standing spine X-ray. Curvatures are further sub-classified by the Lenke classification, based on the type of curve, the type of transition from the pelvis upward, and the degree of alignment in the sagittal plane [2]. While minor curves can be treated conservatively, curves that exceed 50–60 degrees are frequently treated by instrumented spinal fusion surgery. While these surgeries are invasive, they offer a durable correction and generally acceptable long-term outcomes [3,4,5].

Surgical site infection (SSI) following posterior spinal fusion (PSF) for adolescent idiopathic scoliosis (AIS) is one of the most frequently reported complications, ranging in incidence from 1 to 4% [6]. The most common causative agents for SSI in the AIS population are skin flora such as *Staphylococcus aureus* and *Staphylococcus epidermidis* [7]. A recent study of 21 patients undergoing PSF, in which cultures were taken from the surgical site at various stages of the surgical procedure, describes a pattern of residual skin flora spreading to deeper parts of the wound in the course of the surgical procedure [8]. This showed a higher rate of positive cultures from deep within the surgical bed prior to wound closure than immediately following surgical exposure or routine preoperative skin preparation. Overall, 62% of the patients had positive cultures, 90% of which were caused by *C. acnes.* All cultures were sensitive to cefazolin and vancomycin. In addition to the spread of bacteria from the wound edge, recent research has also implicated blood-borne seeding of sites of tissue trauma by bacteria originating at sites naturally colonized by bacteria, such as the nares [9].

Cefazolin is often used for prophylaxis because of its efficacy against skin flora and common Gram-negative bacteria. It is bacteriostatic at low concentrations and bactericidal at higher concentrations. The efficacy of cefazolin depends on its dosing scheme as well as tissue penetrance. Specifically, it is suggested that the dosing should achieve antibiotic concentrations above the minimal inhibitory concentration (MIC) for the targeted bacteria for 90% to 100% of the dosing interval [10,11,12]. Based on the idea that the MIC describes the bacteriostatic effect of cefazolin and the finding that the antibacterial effect of cefazolin increases further for concentrations up to four to five times MIC [13], some even recommend higher concentrations of up to fourfold above MIC. There is, however, limited evidence to guide cefazolin dosing that achieves bactericidal tissue concentrations during the operative phase of care. Recommended individual cefazolin doses range from 1 to 2 g or 30 mg/kg and recommended re-dosing intervals range from 2 to 4 h. Posterior spinal fusion procedures present the additional challenge of fluctuations in drug concentration independent of the dosing scheme due to potentially large volume blood loss, fluid shifts, and significant volume resuscitation.

To study how cefazolin dosing affects cefazolin tissue concentrations during the operative phase of care, we performed a randomized, controlled, prospective pharmacokinetic (PK) pilot study. We measured plasma cefazolin concentrations and used a clinical microdialysis technique to measure skeletal muscle and subcutaneous adipose tissue concentrations of cefazolin given by either intermittent bolus or continuous infusion in patients undergoing PSF for AIS. As a measure of treatment effect, we also calculated the duration of target concentration attainment for unbound cefazolin concentrations at target sites for methicillin-sensitive *Staphylococcus aureus* (MSSA) and Gram-negative bacteria in these two patient groups. We hypothesized that continuous infusion dosing would achieve better target concentration attainment and thus offer improved prophylaxis against SSI pathogens than intermittent bolus dosing.

## 2. Materials and Methods

The study was approved by the University of Florida Institutional Review Board (IRB201701129) and registered with ClinicalTrials.gov (NCT03190668). All subjects provided written consent before randomization. A CONSORT diagram is provided in Supplementary Materials (Figure S1).

### 2.1. Patient Selection and Data Collection

All patients undergoing PSF for AIS at the University of Florida were screened on a rolling basis. Inclusion criteria included a diagnosis of idiopathic scoliosis with planned fusion of at least 6 vertebral levels and age between 12 and 20 years. Candidates were excluded for known allergy to cefazolin, known renal or hepatic insufficiency or failure, abnormalities that precluded insertion of microdialysis catheters into paraspinal muscles, or American Society of Anesthesiologists Physical Status 3 or higher. Data collected from patients included age, sex, height, body mass index, baseline renal function, intraoperative urine output, blood loss, amount of intravenous albumin received during surgery, total allogeneic and autologous transfusion amounts, total intraoperative dose of cefazolin, and any other co-medications received during surgery. In addition, information about Cobb angles, levels fused, surgical instrumentation, and postoperative wound complications was collected.

### 2.2. Anesthesia Management

Our institution adopts a standardized clinical pathway for patients undergoing PSF for AIS [14]. The basic anesthetic technique consists of a total intravenous anesthetic utilizing propofol and remifentanil infusions to facilitate neuromonitoring, administration of tranexamic acid as a bolus dose followed by an infusion and a single dose of methadone prior to incision. Anesthetic drug effect is monitored by electroencephalography with the aim of attaining a continuous tracing or a Bispectral Index Score of 35–60. Details of premedication, airway management, and emesis prophylaxis [15] are left to the discretion of the anesthesia team. The attending anesthesiologist directs all aspects of the intraoperative anesthetic, including adherence to the protocol, intraoperative fluid management, and need for transfusion.

### 2.3. Study Protocol

Patients underwent simple randomization to one of 2 antibiotic dosing regimens: intermittent bolus or continuous infusion. Subjects randomized to bolus dosing were given 30 mg/kg cefazolin IV prior to surgical incision, and treatment was repeated every 3 h for the duration of the operation, reflecting current institutional practice. Subjects randomized to continuous infusion were administered the same initial bolus dose of 30 mg/kg before incision, followed by a continuous infusion of 10 mg/kg per hour until the end of surgery.

### 2.4. Data Collection and Analysis

**Figure 1:**
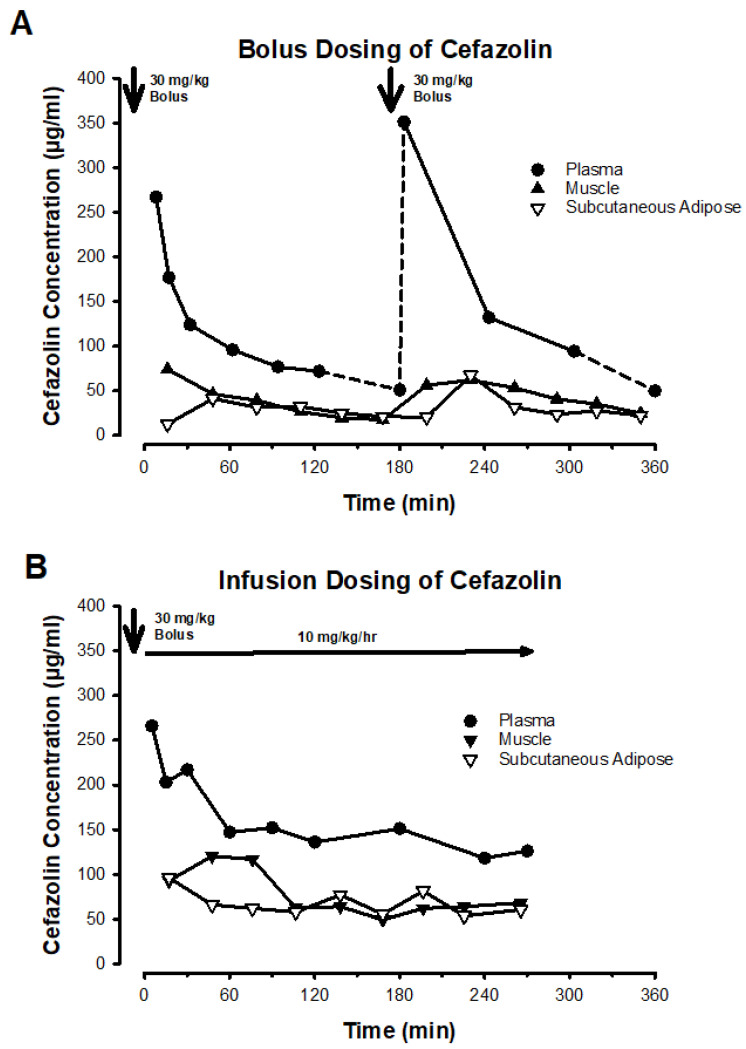
Representative examples of dosing and sampling for the two experimental groups. Panel A shows a patient from the bolus group, with the time of cefazolin administration indicated by a downward arrow. Plasma samples reflect the total cefazolin concentration at the time of sampling, with the exception of the concentration immediately prior to the next bolus. This concentration was derived from the terminal half-life of cefazolin from the last dose, a fact emphasized in the figure by linking it to the concentration-time curve with a dashed line. Interstitial cefazolin concentrations from muscle and subcutaneous adipose samples reflect the average free concentration over the 30 min microdialysis sampling period. Panel B shows a patient from the continuous infusion group.

Plasma samples were obtained from an arterial catheter in both groups. Samples were drawn prior to the original dose and after first cefazolin administration at 5 min, 15 min, 30 min, 60 min, 90 min, 180 min, and every 60 min thereafter. Blood samples were centrifuged at 3000 rpm for 15 min and plasma was transferred. Serial microdialysis samples were obtained from skeletal muscle and adipose tissue near the surgical site in 30 min intervals throughout the surgery. Samples were stored at −80 °C until analysis. Both plasma samples and microdialysis samples were analyzed by a previously published high-performance liquid chromatography–tandem mass spectrometry method [16]. Figure 1 depicts a representative patient example from each group to illustrate dosing and sampling.

### 2.5. Clinical Microdialysis Procedure

**Figure 2:**
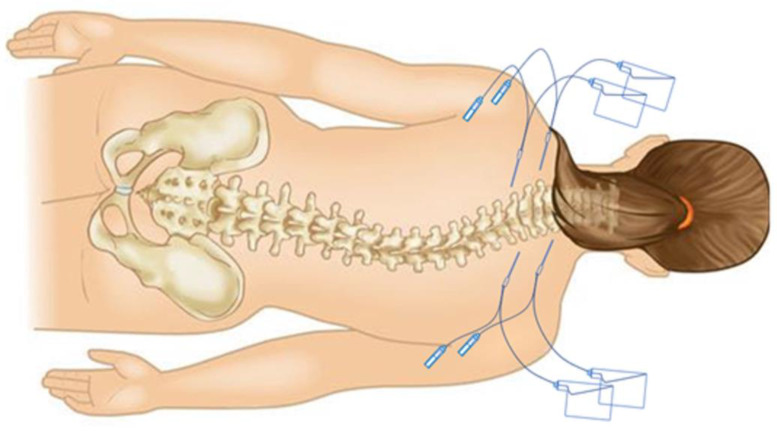
Schematic illustration of the microdialysis technique and the probe placement. On either side of midline one coaxial dialysis probe was placed in subcutaneous adipose tissue and one in the paraspinous muscles. Probes were perfused by elastomeric pumps and perfusate was collected in sample vials.

Two coaxial microdialysis probes were inserted percutaneously after the induction of anesthesia into left and right paraspinal muscles 2 levels superior to the planned incision, and 2 additional probes were inserted into subcutaneous adipose tissue lateral to the muscle probes (Figure 2). Each microdialysis catheter contained a 30 mm polyarylethersulfone membrane with a molecular cutoff of 20 kDa (63 MD catheter; M Dialysis AB, Solna, Sweden) and was connected to a 107 microdialysis pump (M Dialysis AB) with a perfusion flow rate at 1 µL/min. All 4 probes were perfused with normal saline containing 10 µg/mL cefuroxime prepared by the investigational drug service as a calibrator for clinical microdialysis of cefazolin [15]. The interstitial concentration of cefazolin was calculated using the following equation:

C_cefazolin, tissue_ = 100 × (C_cefazolin, dialysate_/Cefazolin Recovery %),

where cefazolin recovery is proportionate to the rate of cefuroxime disappearance from the perfusate as expressed in the following equation:

Cefazolin Recovery % = 100 × (Ccefuroxime, perfusate − Ccefuroxime, dialysate)/Ccefuroxime, perfusate.

### 2.6. Pharmacokinetic Analysis

Key pharmacokinetic parameters (C_max_, t_max_, t_1/2_, CL, AUC) for cefazolin were determined from concentration-time profiles in plasma, subcutaneous adipose tissue, and muscle tissue using a non-compartmental method conducted in commercially available software (Phoenix WinNonlin, Pharsight Corporation, CA, USA). For PK parameter calculations of plasma cefazolin concentrations after intermittent bolus dose administration, we extrapolated missing concentrations at the nadir and peak of the PK disposition curve after the second bolus dose using the terminal half-life of cefazolin during the last dosing interval and the 5 min post-dose concentrations from the previous dose, respectively. The biologically active free plasma concentrations of cefazolin were only measured in the first 4 subjects and calculated based on the average protein binding for the remaining subjects. Due to the nature of microdialysis collection, the C_max_ values for the interstitial concentrations of cefazolin represent the average concentration throughout the 30 min collection interval. Plasma and tissue concentrations of cefazolin were related to published MIC values to determine the time above MIC (*f*T > MIC), which is the pharmacokinetic–pharmacodynamic (PK-PD) factor most closely related to the antibacterial effect of cefazolin in vivo.

### 2.7. Statistical Analysis

Data were checked for implausible values and distributional form. Group comparisons were performed using Wilcoxon rank sum tests. The detectable effect size for the pilot study (assuming 6 patients in each group) was 1.8 times the standard deviation of a continuous measure (i.e., AUC of total drug exposure) at a 0.05 level of significance and with 80% power. Tests of hypotheses were 2-sided using a specified significance level of 0.05. SAS software version 9.4 (Cary, NC, USA) was used for data analysis.

## 3. Results

### 3.1. Population Characteristics

Thirteen patients were enrolled in this study, with twelve randomized to either bolus or continuous infusion dosing. Six patients were included in each of the groups. One patient was withdrawn because study staff were not available at the time of the procedure. Cefazolin tissue concentration data were found to be unusable for three patients due to problems with sample processing yielding analyzable data for a total of nine patients (four patients in the bolus group and five patients in the infusion group). Demographic, procedural, and postoperative data are presented in Table 1. Patients were well matched for health status, weight, renal function, intraoperative blood loss, and fluid resuscitation. Half of the patients in the bolus group were male, while all in the infusion group were female. The bolus and infusion groups did not differ in the number of patients receiving adjunctive medications. Most received lidocaine and fentanyl at induction and acetaminophen for supplemental analgesia. One patient of four in the bolus group and two out of five in the infusion group did not receive midazolam as an anxiolytic. No participants received allogeneic red blood cells intraoperatively. Surgeries were typically concluded in under 6 h. Only one patient in the bolus group required a third dose of cefazolin during closure of the operative incision. This dose was excluded from analysis.

### 3.2. Drug Dosing and Cefazolin Concentrations in Plasma, Muscle, and Subcutaneous Adipose Compartments

**Figure 3:**
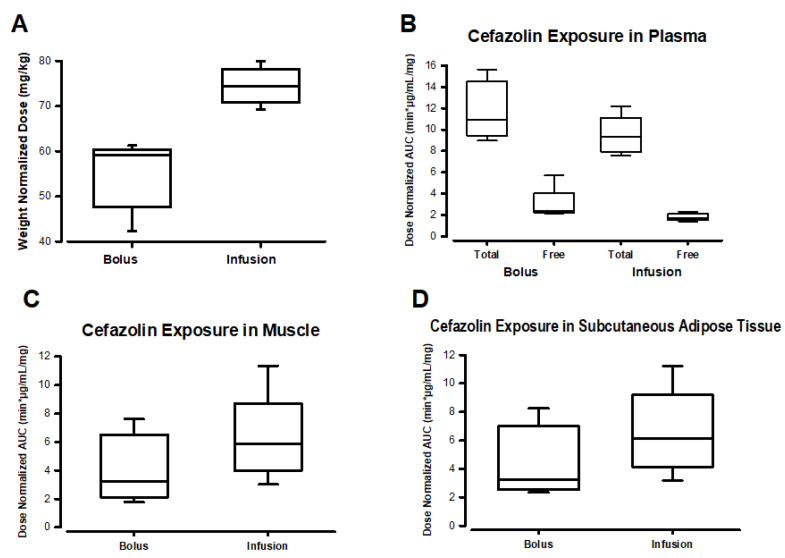
Drug exposure for bolus and infusion dosing of cefazolin. Panel (**A**) depicts the administered drug dose normalized to body weight. Panel (**B**): total drug exposure in plasma expressed as area under the concentration-time curve (AUC) normalized by dose and resulting exposure to unbound cefazolin. Panel (**C**) total drug exposure (dose-normalized AUC) in muscle. Panel (**D**) total drug exposure (dose-normalized AUC) in subcutaneous adipose tissue.

As expected from the dosing scheme, patients in the infusion group received more cefazolin than those in the bolus group (Figure 3A, Table S1). This is because the infusion group received both an IV bolus and an equivalent infusion dose immediately after, which added an additional cefazolin dose to the total dose administered in the infusion group. When analyzing total drug exposure as area under the concentration-time curve (AUC) of plasma concentrations, the AUC was similar in both groups for both total and free cefazolin after dose normalization (Figure 3B, Table S1). However, the total drug exposure (AUC) in muscle and subcutaneous adipose tissue was greater in the infusion group, even after values were normalized by the administered cefazolin dose (Figure 3C,D, Table S1). No statistically significant differences were found between groups in comparing normalized doses or AUC values (Table S1).

**Figure 4:**
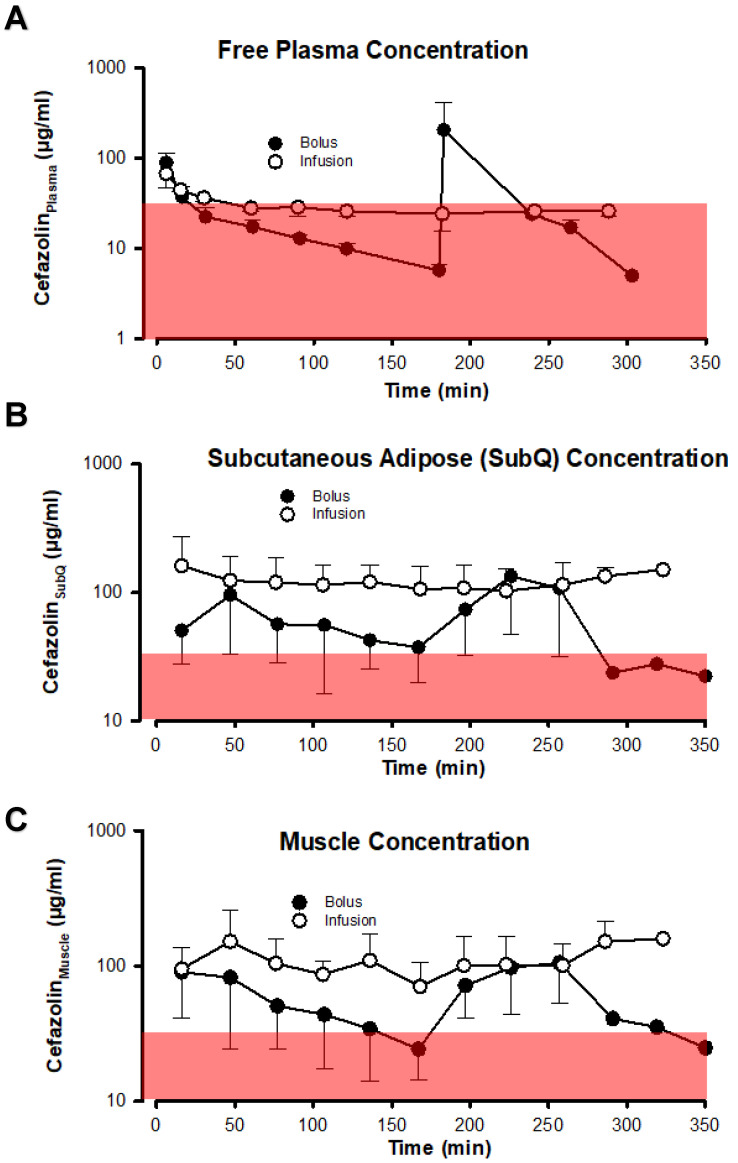
Concentration-time curves of cefazolin in the bolus and infusion groups in plasma (Panel (**A**)), muscle (Panel (**B**)) and subcutaneous adipose tissue (Panel (**C**)). The area shaded in red represents a cefazolin concentration of less than 32 µg/mL to illustrate the effect of variability in cefazolin concentrations between the two dosing regimens.

**Figure 5:**
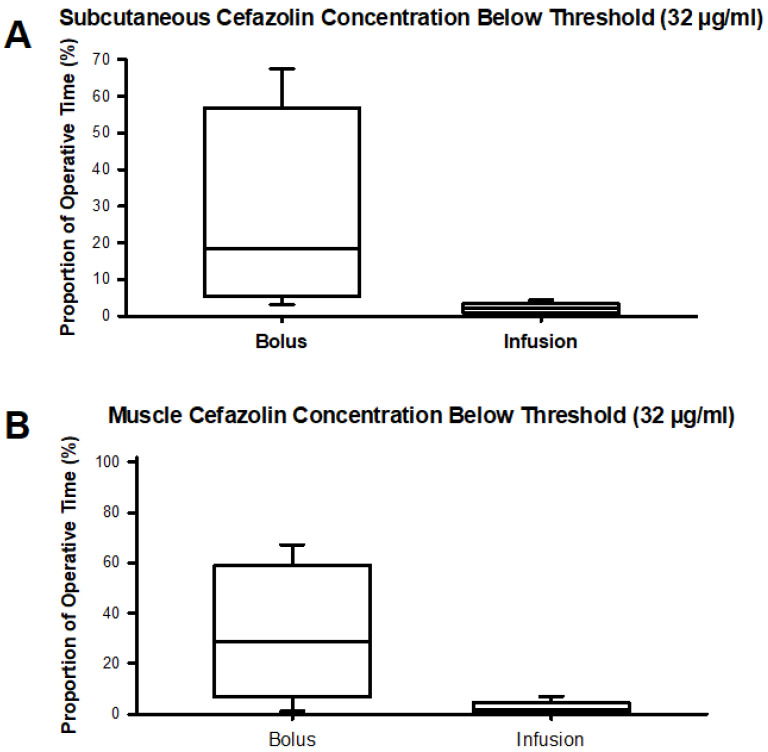
Proportion of the operative time spent below a threshold concentration of 32 µg/mL cefazolin in adipose (Panel (**A**)) and muscle (Panel (**B**)) compartments for bolus and infusion groups.

The antibacterial effect of the two different dosing schemes based on unbound cefazolin concentrations in plasma, muscle, and subcutaneous adipose tissue is depicted in Figure 4 using the most relevant PK-PD metric *f*T > MIC. Bolus dosing of cefazolin resulted in much more variable and typically lower cefazolin concentrations in both adipose tissue and muscle compartments than infusion dosing. More importantly, using a cefazolin concentration threshold of 32 µg/mL, chosen to exceed by fourfold a bacteriostatic concentration of 8 µg/mL that is based on the typical serial dilution procedure used in determining minimal inhibitory concentrations of cefazolin to prevent growth of bacteria (Table 2) in 90% of cases, shows that plasma concentrations generally decreased below 32 µg/mL in both the bolus and infusion groups. However, as infections usually occur in tissue rather than in plasma, it is more critical to evaluate the PK-PD metric in tissue. In subcutaneous adipose tissue and muscle, the variability in cefazolin concentration introduced by bolus dosing was sufficient to drive tissue concentrations below 32 µg/mL for a period preceding the next bolus dose. Figure 5 (Table S2) depicts the time spent below a cefazolin concentration of 32 µg/mL in subcutaneous adipose tissue and muscle tissue. For the bolus group, median times spent below the 32 µg/mL threshold in muscle approximate a third of the typical operative time of 6 h. Free plasma concentrations decreased below 32 µg/mL regardless of the cefazolin dosing scheme in most patients and for the majority of the procedure. No statistically significant differences were found between groups in comparing time spent below a cefazolin concentration of 32 µg/mL (Table S2).

## 4. Discussion

Our study found that continuous infusion of cefazolin more consistently resulted in bactericidal concentrations in subcutaneous and muscle tissues than cefazolin administration by intermittent bolus in patients undergoing PSF for AIS at an experienced tertiary care center. Particularly for the muscle compartment, tissue concentrations were less than a target of 32 μg/mL for nearly a third of the entire procedure when cefazolin was administered as an intermittent bolus. While tissue concentrations are an intermediate outcome, they lie on the causal chain connecting skin flora to surgical site infection. Given the low rate of SSIs in this relatively healthy cohort of immunocompetent patients, a definitive outcome study would require a sample size that well exceeds the capacity of a single center, and indeed we found neither wound complications nor SSIs. Although our study was not designed to study SSI directly and was underpowered to yield statistically significant data, we believe it offers several important insights into how to better dose cefazolin in this population.

Our study compared cefazolin infusion dosing with a bolus dosing regimen that is relatively aggressive in both the dose and in the redosing interval. Therefore, both regimens are likely to cover Gram-positive organisms such as MSSA and *S. epidermidis* adequately throughout the surgical period, especially since even during treatment of a Gram-positive infection, tissue concentrations above MIC are only required for 40% to 50% of the time [13]. Similar results to ours for plasma and muscle concentrations were reported for bolus administration of cefazolin in PSF for AIS [17]. For the overall less susceptible and more variably susceptible Gram-negative organisms (Table 2), the impact of the dosing scheme is likely more important. This is reflected in the recommendation to achieve target concentrations in excess of the MIC of Gram-negative organisms at the site of infection for a minimum of 60% to 70% of the treatment time [13]. Whether the paradigm of “treatment of an active infection” is applicable to the perioperative period is unknown, but a study of SSIs in 228 spine fusion procedures identified under-dosing of cefazolin perioperatively as an important risk factor for both Gram-positive and -negative SSIs [18].

Surgical site infections are likely multifactorial. Some infections are clearly caused by direct contamination, i.e., the spread of residual skin flora remaining after antisepsis [8]. For other SSIs, the patient’s own microbiome provides the causative organism when combined with the immunologic perturbation and disruption of physiologic bacterial communities of the perioperative period [9]. For example, neutrophils may transport viable *S. aureus*, internalized at a colonized site such as the nares, to a site of tissue trauma such as the surgical incision. For both types of infections, consistent attainment of adequate tissue concentrations should reduce active infections of the treated patient specifically and the development of bacterial resistance in general.

Our finding that infusion dosing minimized the variability of measured drug concentrations (typically measured in plasma) is consistent with the findings from other studies that compared infusion with bolus dosing perioperatively [11,19]. Similarly, infusion dosing of cefazolin is preferred in settings such as the treatment of infective endocarditis [20] or bone/joint infections [21], where the maintenance of bactericidal free plasma concentrations is desirable. More importantly, in cardiac surgery, where cardiopulmonary bypass and bleeding affect active tissue concentrations, cefazolin dosing by continuous infusion reduced the rate particularly of superficial SSIs by nearly fourfold [22]. This improvement in outcome is consistent with the finding of increased infection with underdosing of cefazolin in pediatric non-AIS spine fusion [18], which shares with cardiac surgery the characteristics of greater blood loss, fluid shifts, and invasiveness.

There are several limitations to our study. First, the study is not sufficiently powered to demonstrate statistical significance in any differences between the two groups. Therefore, even though the measurements within each subject were internally consistent and the overall results fit with those in comparable clinical situations, there remain considerable sources of bias in the final interpretation of our results. The data demonstrate a clear trend toward greater concentrations in the infusion group, suggesting that bactericidal concentrations are more consistently achieved via continuous infusion. Such bactericidal concentrations may be warranted with implantation of hardware and organisms that are known for their ability to form biofilms. Second, our study is focused on measuring tissue concentrations and pharmacokinetics, which are intermediary to the outcome of interest, SSI. Third, consistent with others [23], we find no effect of blood loss on cefazolin tissue distribution. Thus, even though our measurement techniques could be easily adapted, we are unsure to what extent our data generalize to neuromuscular scoliosis surgeries, where intraoperative blood loss and transfusion are more prevalent.

Future research should evaluate the effect of infusion versus bolus dosing on the ultimate outcome, the rate of SSIs. To achieve adequate power, such a study will likely involve many institutions. A second future direction should be mechanistically oriented. We find our technique of sampling from relevant compartments for SSI integrates relatively easily into the complex sequence of corrective surgery for scoliosis and provides useful pharmacokinetic data. It would be of great interest to apply our sampling technique to patients undergoing surgery for neuromuscular scoliosis, not only because the rate of SSIs is greater in that population, but also because intraoperative fluid shifts are greater and transfusion rates are higher.

## 5. Conclusions

Our study demonstrates that the administration of cefazolin by continuous infusion intraoperatively results in higher plasma, subcutaneous adipose tissue, and muscle concentrations of cefazolin in healthy patients undergoing PSF for AIS than cefazolin administration by intermittent bolus. Continuous infusion is more likely to result in antibiotic concentrations that are bactericidal and should reduce the likelihood of surgical site infections in this population. Our technique of combining microdialysis with simultaneous retro-dialysis of a calibrator molecule with similar chemical structure integrates relatively easily into the logistics of a complex surgical procedure.

## Figures and Tables

**Table 1:** Demographic and Intraoperative Characteristics of the Study Groups.

		Bolus Group (*n* = 4)	Infusion Group (*n* = 5)
**Age, years (mean [SD], range)**		16 (1) (13–17)	13 (1) (12–15)
**Female (%)**		25	100
**BMI (kg/m^2^, mean [SD])**		20 (2)	24 (7)
**Duration of Surgery** **(min, mean [SD])**		284 (64)	302 (36)
**Creatine Clearance** **(ml/min, mean [SD])**		124 (44)	194 (101)
**Fluid Balance (ml, mean [SD])**	Blood Loss	825 (263)	640 (230)
	Urine Output	1050 (897)	1274 (747)
	Crystalloid	3400 (860)	3237 (1039)
	Colloid	563 (427)	500 (177)
	Autologous Blood	306 (240)	40 (89)
**Surgical Details**	Cobb Angle	58 (10)	74 (10)
	Levels Fused (number, SD)	9 (3)	12 (1)
	Additional Procedures	Ponte Osteotomies (3–4 levels)	Ponte Osteotomies (3–5 levels)
**Infectious Complications**	Wound Complications	None	None
	Surgical Site Infection	None	None

Abbreviation: BMI, body mass index. SD, standard deviation.

**Table 2:** Minimal Inhibitory Concentrations of Cefazolin for Typical Bacterial Pathogens ^1^.

Pathogen		Inhibitory Concentrations (µg/mL)			Sensitivity of Isolates (%)		
		MIC_50_	MIC_90_	Range	Susceptible	Intermediate	Resistant
GP	*Staph. aureus (MSSA)*	<0.5	1	<0.06 to 4	Generally considered susceptible		
GP	*Staph. epidermidis*	1	4	<0.05 to >128	Generally considered susceptible		
GN	*Escherichia coli*	2	>128	<0.5 to >128	58	14	28
GN	*Klebsiella pneumoniae*	2	>128	1 to >128	79	8	14
GN	*Proteus mirabilis*	4	8	2 to 32	2	60	38

Abbreviations: GP = Gram-positive; GN = Gram-negative; MIC = minimum inhibitory concentration (i.e., the lowest concentration of an antibacterial agent expressed in mg/L [μg/mL] which, under strictly controlled in vitro conditions, completely prevents visible growth of the test strain of an organism [from pathogens 2021]); MSSA = methicillin-sensitive *Staphylococcus aureus*; Staph. = Staphylococcus. ^1 ^ Source: Canadian Antimicrobial Resistance Alliance. CANWARD pathogens. Available online: http://www.can-r.ca/study.php?study=canw2021&year=2021 (accessed on 11 April 2024).

## Data Availability

The raw data supporting the conclusions of this article will be made available by the authors on request.

## Supplementary Materials

The following supporting information can be downloaded at: https://www.mdpi.com/article/10.3390/jcm13123524/s1, Figure S1: CONSORT Diagram; Table S1: Comparison of Cefazolin Concentrations; Table S2: Proportion of the Operative Time Spent Below a Threshold Concentration of 32 µg/mL Cefazolin in Adipose and Muscle Compartments for Bolus and Infusion Groups.

## Dedication

This work is dedicated to the late Hartmut Derendorf, PhD, Distinguished Professor of Pharmaceutics, University of Florida College of Pharmacy.
