# Supplementary material for: Dosing Cefazolin for Surgical Site Infection Prophylaxis in Adolescent Idiopathic Scoliosis Surgery: Intermittent Bolus or Continuous Infusion?—A Pilot Study
Source: J Clin Med. 2024 Jun 16;13(12):3524. doi: 10.3390/jcm13123524 (PMC11204537; doi:10.3390/jcm13123524)
Supplement: Supplementary file 1 [file jcm-13-03524-s001.zip › Table S1.pdf]

Table S1. Comparison of Cefazolin Concentrations.

	**Bolus**<br>***Median (Min, Max)***<br>***Mean (SD)***	**Infusion**<br>***Median (Min, Max)***<br>***Mean (SD)***	***p* value**
Normalized Dose (mg/kg)	60.33 (59.17, 89.95)<br>67.45 (15.03)	73.26 (64.71, 88.12)<br>74.97 (9.33)	0.3024
Plasma Cefazolin Concentration (min g/ml/mg)	8.96 (7.56, 15.64)<br>10.18 (3.26)	8.23 (7.26, 12.17)<br>9.07 (2.05)	0.6859
Muscle Cefazolin Concentration (min g/ml/mg)	3.22 (1.77, 7.62)<br>3.96 (2.53)	5.84 (3.01, 11.35)<br>6.23 (3.10)	0.4162
Subcutaneous Cefazolin Concentration (min g/ml/mg)	3.21 (2.33, 8.24)<br>4.25 (2.69)	6.13 (3.20, 11.22)<br>6.56 (2.99)	0.3024

Abbreviations: Min, minimum; Max, maximum; SD, standard deviation
