# Supplementary material for: Dosing Cefazolin for Surgical Site Infection Prophylaxis in Adolescent Idiopathic Scoliosis Surgery: Intermittent Bolus or Continuous Infusion?—A Pilot Study
Source: J Clin Med. 2024 Jun 16;13(12):3524. doi: 10.3390/jcm13123524 (PMC11204537; doi:10.3390/jcm13123524)
Supplement: Supplementary file 1 [file jcm-13-03524-s001.zip › Table S2.pdf]

Table S2. Proportion of the Operative Time Spent Below a Threshold Concentration of 32 μg/ml Cefazolin in Adipose and Muscle Compartments for Bolus and Infusion Groups.

	**Bolus** *Median (Min, Max)* *Mean (SD)*	**Infusion** *Median (Min, Max)* *Mean (SD)*	***p* value**
Adipose	.18 (.03, .67)<br>.27 (.29)	.02 (.01, .04)<br>.02 (.01)	.0709
Muscle	.29 (.01, .67)<br>.32 (.28)	.02 (.01, .07)<br>.03 (.03)	.2148
Plasma	.68 (.64, .80)<br>.71 (.07)	.84 (.63, .85)<br>.80 (.09)	.1777

Abbreviations: Min, minimum; Max, maximum; SD, standard deviation
