# Supplementary figures and images for: Dosing Cefazolin for Surgical Site Infection Prophylaxis in Adolescent Idiopathic Scoliosis Surgery: Intermittent Bolus or Continuous Infusion?—A Pilot Study
Source: J Clin Med. 2024 Jun 16;13(12):3524. doi: 10.3390/jcm13123524 (PMC11204537; doi:10.3390/jcm13123524)

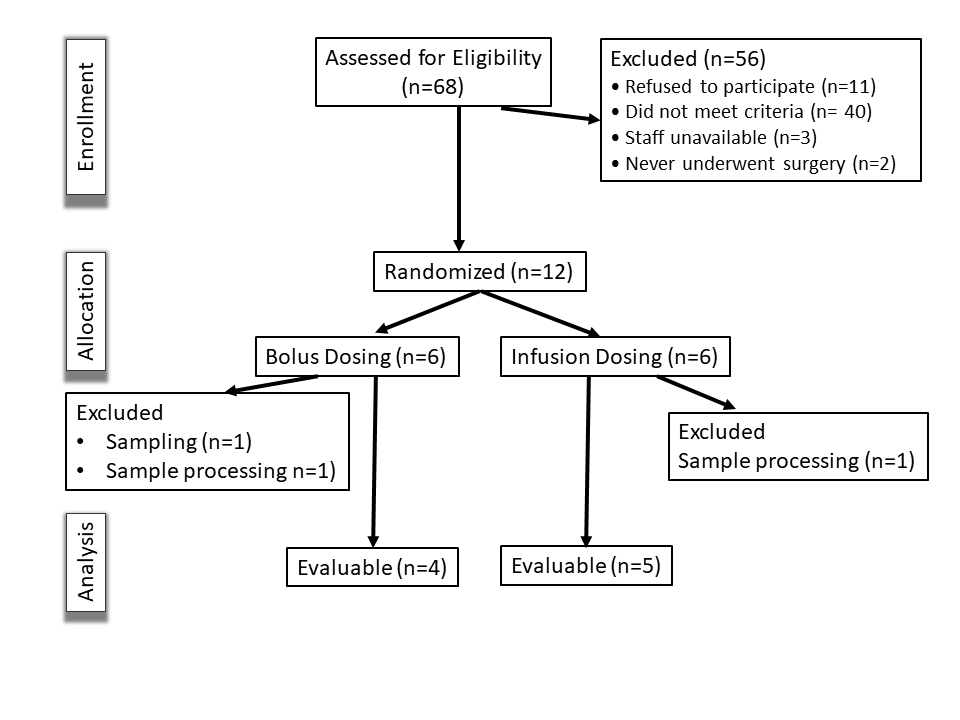

Supplement: Supplementary file 1 [file jcm-13-03524-s001.zip › Figure S1.tif]
